# Soil bacterial and fungal communities respond differently to the biochar amendment in a cadmium-contaminated paddy field

**DOI:** 10.1038/s41598-025-02182-w

**Published:** 2025-10-14

**Authors:** Min Zhang, Jianwu Jiang, Qijie Yin, Lan Ding, Xiaoli Yuan, Yonggen Chen, Wenbo Liu, Bin Guo, Shengdao Shan

**Affiliations:** 1 Zhejiang Key Laboratory of Recycling and Eco-Treatment of Waste Biomass, Zhejiang University of Science and Technology, No.318 Liuhe Road, Hangzhou, 310023 Zhejiang People’s Republic of China; 2 Lin’an A&F Bureau, Hangzhou, 311300 People’s Republic of China; 3 Zhejiang Academy of Agricultural Sciences, Hangzhou, 310021 People’s Republic of China

**Keywords:** Soil microbial community, BCR, Paddy field, Biochar amendment, Cadmium fractionation, Soil microbiology, Environmental sciences

## Abstract

For slightly and moderately cadmium (Cd)-contaminated paddy soils, in situ stabilization technologies such as biochar amendment provide a sustainable way of safe agricultural production. A two-year study was conducted to investigate soil microbial communities responses to biochar amendment in a slightly Cd-contaminated paddy field. Soil Cd fractions, organic carbon content, microbial communities, and their relationships were analyzed. The results showed that biochar application decreased Cd accumulation in brown rice by 13.56–24.56% and HOAc-soluble Cd content by 35.65–52.5%. It mainly induced the transformation of soluble Cd to oxidizable and residual Cd. Soil organic carbon (TOC), total nitrogen (TN), available potassium (AK) and available phosphorus (AP) contents tended to increase with biochar amendment. Biochar application significantly increased bacterial diversity but had no significant effect on fungal diversity. Soil oxidizable Cd contents were the most influencing environmental factors of bacterial communities, whereas soil pH were the most influencing environmental factors of fungal communities. These results indicate that the fungal community structure was influenced mainly bychanged soil pH, whereas the bacterial community structure was influenced by biochar application mainly due to Cd immobilization.

**Supplementary Information:**

The online version contains supplementary material available at 10.1038/s41598-025-02182-w.

## Introduction

Among all toxic metals, cadmium (Cd) attracts more attention due to its high mobility, strong toxicity, and various pollution pathways. As Cd is readily absorbed by rice, paddy soil Cd contamination has been a concern, especially in the rice production area of South China^1–4^. Many technologies have been developed to remediate Cd-polluted soils. Organic additives (OAs), such as biochar, used for in situ Cd stabilization is environmentally friendly and highly efficient for agricultural Cd pollution remediation^5–7^. Biochar has been widely used in soil heavy metal immobilization due to its porous structure, high pH value, rich functional groups and high carbon content^8–10^. Biochar immobilizes toxic metals in the soil through ion exchange, complexation by functional groups, physical adsorption, and precipitation. Rathnayake et al.^11^ reported that biochar, especial aged biochar can significantly reduce exchangeable Cd fraction in soil and increased the reducible Cd fraction in soil. Biochar application not only can reduce the mobility of heavy metal in but also improve soil structure and buffering capacity^9,10^. Moreover, biochar is expected to have long-lasting remediation effects due to its highly aromatic structure and relative recalcitrant character in soil. long-term biochar application changed the soil environment and carbon substrate quality, and then influence soil microbial activity directly or indirectly^6,12,13^.

Previous studies investigated the influences of biochar feedstock and application amount on cadmium immobilization in soil and its accumulation in rice grains, as well as soil properties and rice growth^14–16^. It is commonly reported that biochar application can significantly reduce available Cd contents in soil and total content in rice. On the other hand, the Cd immobilized by biochar may become mobile over time. Studies found that biochar addition has a stronger effect on Gram-negative bacteria than on Gram-positive bacteria^17–19^. The effect of biochar application on soil microbial community composition is mainly due to the changes in soil pH and C/N ratio. With the aging of biochar in soil and the dissolution of organic carbon^20^, the specific surface area of biochar decreases^21^, which would lead to changes in Cd immobilization and microorganism communities in the biochar amended fields^21,22^. Microbe can induced carbonate precipitation in paddy soil, leading to Cd immobilization by bacteria. It enhanced the formations of oxidizable and residual Cd to reducing Cd mobilization. It was suggested that the evolution of functional microbial communities shaped by soil amendments, thereby reducing Cd uptake by crops^23^. Mei et al.^24^ further explored that contribution of the reduced HOAc Cd content in biochar amended soil was highest towards improved bacterial and fungal alpha diversity. They also found significant changes in Bacillus, Streptomyces, Arthrobacter and Aspergillus, appeared to be key factors during the stabilization of Cd fractions.

Despite previous works, the effect of biochar application on Cd fractionation and the relationship between soil microbes and soil environmental factors in Cd-contaminated soils remains largely uninvestigated. To this end, biochar was applied at four levels in a slightly Cd-contaminated paddy field, which was located in the downstream area of a tungsten mine and had been irrigated with nearby stream polluted by mine wastewater for a long time. Soil bacterial, fungal, and archaea communities were characterized via 16S rRNA gene sequencing. In addition, changes in soil dissolved organic carbon (DOC) and Cd fractions were studied. The objective is to explore (1) the effect of biochar amendment on soil Cd reduction and transformation of Cd fractions within 2 years and (2) the relationship between Cd fractions in soil and bacterial / fungal communities.

## Materials and methods

### Experimental site and soil

The experimental field (N 29° 56′, E 119° 13′, 400 m a.s.l.) located about 8 km downstream of a tungsten mine in the northwest of Zhejiang Province, China. Though the mine has closed since 2000s, wastewater discharged still brought heavy metal accumulation in downstream agricultural soils by polluted irrigation water. It induced excessive heavy metals in local agricultural products. The experimental site has a subtropical monsoon climate. The soil in the field is a Ferralic Acrisols. Heavy metal contents of the soil are listed in Table 1^15^. According to the "Soil Environmental Quality—Risk Control Standard for Soil Contamination of Agricultural Land (Trial)" (GB 15618-2018) in China, for paddy fields, when the soil pH is in the range of 5.5–6.5, the risk screening value of cadmium is 0.4 mg/kg, and the risk control value of cadmium is 2.0 mg/kg. Maize straw biochar used in the study and had a specific surface area of 57.68 m^2^ g^−1^.

**Table 1:** Soil heavy metal contents (mg kg^−1^) in the experimental site.

Cd	Pb	Ni	Cr	Zn	Cu
0.70 ± 0.1	16.59 ± 3.2	7.10 ± 1.4	21.76 ± 3.5	81.98 ± 10.2	15.95 ± 2.3

### Experimental design and sampling

Four levels of biochar were set up in triplicate: 0 (CK), 7.5 (C1), 15 (C2), and 30 t ha^−1^ (C3). The experiment adopted a completely randomized block design. The plots were 50 m^2^ in area and separated by 0.5 m-wide buffer rows. Each plot had an irrigation inlet and a drainage outlet. Biochar was mixed with the surface soil before rice transplanting in 2015. Late japonica rice was planted in June and harvested in October in both 2015 and 2016. After rice harvest in 2016, Soil (0–10 cm) were sampled three points in each plot and then mixed into one composite sample. A portion of the fresh soil sample was frozen at − 80 °C for later molecular biological analysis, and the left was air-dried and ground for the determination of soil properties including Cd content. At harvest, rice yield of each plot was determined. Rice grains were dried for the analysis of Cd content.

### Soil property determination

Soil pH value was determined by a pH meter (Mettler-Toledo, Switzerland). Soil total Cd was digested with HNO_3_-HF-H_2_O_2_ and plant Cd was digested with HNO_3_-H_2_O_2_. Soil Cd fractionation was performed using the three-step sequential extraction procedure (BCR) to obtain the acid-soluble (HOAc-extractable), reducible (NH_2_OH-HCl-extractable), and oxidizable (H_2_O_2_-NH_4_OAc-extractable) Cd fractions^25,26^. Cd concentration in the extracted solution was measured with an ICP-MS (Optima 8300, Perkin-Elmer, America). For soil DOC determination, 10 g dry soil was extracted with 25 mL H_2_O and shaking at 25 °C for 30 min. The C concentration was determined by TOC analyzer (Shimadzu, Japan). Soil total C and N (TOC, TN) were determined using Elementar CN combustion analyzer (Vario EL, Germany). Soil available N (AN) was determined using KCl extraction. Soil AKwas determined using ammonium acetate extraction and APand was determined using sodium bicarbonate extraction.

### DNA extraction and Illumina MiSeq sequencing

For details, see the supplementary file.

### Construction of co-occurrence networks

Co-occurrence networks of bacterial and fungal communities were constructed based on adjacency matrix theory. To ensure data reliability and reduce spurious correlations, OTU data were filtered by retaining only OTUs with an average relative abundance ≥ 0.01% and occurrence frequency exceeding a defined threshold. The resulting adjacency matrices were then imported into R (v4.3.2) and converted into network-compatible data formats. Finally, network visualisation was conducted using the interactive platform Gephi (https://gephi.org).

### Statistical analysis

Significant differences among treatments were assessed by analysis of variance (ANOVA) followed by LSD tests. The ‘vegan’ package in R (v4.3.2) was used to calculate fungal and bacterial community diversity indices, including Simpson, Chao1, and richness. To evaluate β-diversity, Bray–Curtis distances were calculated using the vegdist() function, and visualised by principal coordinate analysis (PCoA). Pearson correlation analysis was performed to assess relationships between microbial diversity and soil properties. Redundancy analysis (RDA) was further employed to explore the associations between microbial community composition and environmental variables. All figures were generated using the ‘ggplot2’ package (v4.3.2) in R.

## Results

### Plant growth and Cd uptake

Compared to CK, biochar application increased rice yields in two years after application (Table 2). However, it significantly decreased the Cd content in brown rice by 13.65–24.16%, with a larger decrease in C1 than in C2 and C3.

**Table 2:** Rice yield and Cd uptake.

Treatment	Yield (t ha^−1^)	Cd content decrease in brown rice compared to CK
CK	6.6bc^a^	–
C1	7.0b	24.16%
C2	7.0b	14.57%
C3	8.1a	13.65%

^a^Means followed by different letters within a column indicate significant difference between treatments at *P* < 0.05.

### Soil chemical properties and cadmium fractions

**Fig. 1:**
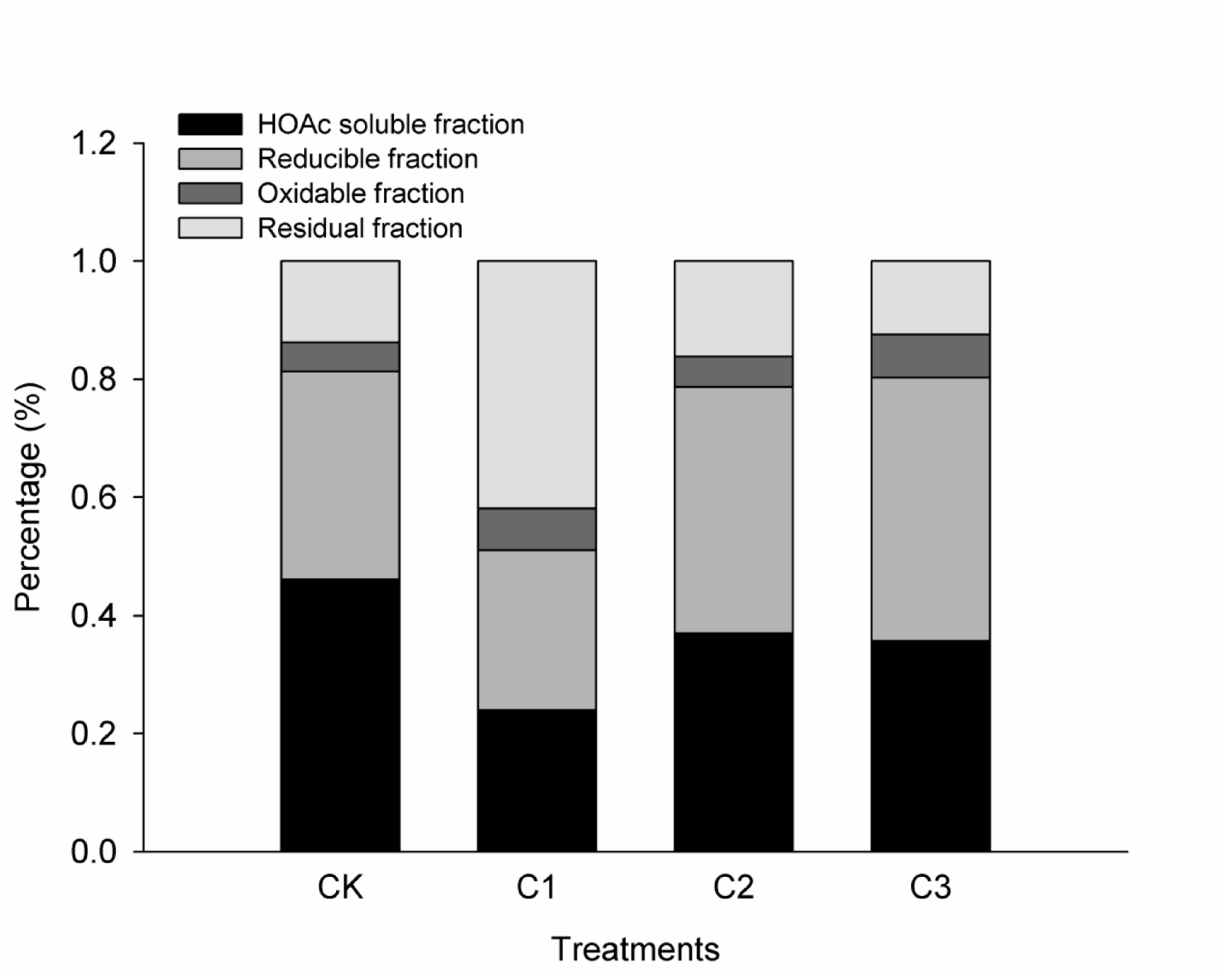
Soil cadmium fractions in different treatments two years after biochar application at different dosages.

Although biochar application tended to increase soil pH, this effect was not significant except at the dosage of C1 (Table 3). Compared to CK, soil total organic carbon (TOC) was significantly higher by 17.5–22.6% in the biochar application treatments. Soil DOC content in C1 was significantly increased, whereas those in C2 and C3 were significantly decreased compared to CK. Soil TN content in C3 was higher than that in CK. There was no significant difference in soil AN between CK and the biochar application treatments. Soil AK contents of the treatments with biochar application more than 15 t ha^−1^ was significantly higher than that of CK. Biochar application also promoted increasing of soil AP content. Compared to CK, the soluble Cd contents in C1, C2, and C3 were decreased by 52.5%, 35.65%, and 46.0%, respectively. Biochar application mainly induced the transformation of soluble Cd to oxidizable and residual Cd (Fig. 1).

**Table 3:** Soil chemical properties in different treatments two years after biochar application at different dosages.

Treatment	pH	TOC (%)	DOC (mg kg^−1^)	TN (%)	AN (mg kg^−1^)	AK (mg kg^−1^)	AP (mg kg^−1^)
CK	5.62b^a^	1.323b	0.124b	0.142b	142.800a	135.556c	13.310b
C1	5.82a	1.554a	0.343a	0.153ab	141.050a	131.667c	26.224a
C2	5.69b	1.576a	0.117c	0.147ab	148.050a	143.333b	16.457ab
C3	5.67b	1.623a	0.078d	0.155a	170.217a	196.667a	22.706b

^a^Means followed by different letters within a column indicate significant difference between treatments at *P* < 0.05.

### Bacterial and fungal abundances and diversity

Averages of 3809, 3709, 3796, 3850 sequences per sample were obtained for the CK, C1, C2, and C3 treatments, respectively, for the bacterial 16S rRNA gene and fungal ITS gene (Table 4). The Chao1 index of soil bacteria and fungi tended to be higher in the biochar application treatments compared to CK, while not significant. The Simpson index of soil bacteria was not significantly different among all treatments. The Simpson index of soil fungi in C1 and C3 was significantly lower than that of CK. The biochar application tended to increase bacterial abundance and fungal alpha-diversity.

**Table 4:** The Simpson and Chao indices of soil bacterial and fungal communities.

Treatment	OTUs	Bacterial community			Fungal community		
		Simpson^a^	Chao1	Richness	Simpson	Chao1	Richness
CK	3809	99.69 ± 0.04ab^b^	1786.6 ± 37.9a	1695.0 ± 51.5a	93.64 ± 0.04b	473.7 ± 153.3a	335.7 ± 72.7ab
C1	3709	99.68 ± 0.04ab	1811.9 ± 49.3a	1746.7 ± 49.1a	90.86 ± 0.04c	559.0 ± 51.0 a	390.3 ± 11.1 a
C2	3796	99.73 ± 0.01a	1783.96 ± 30.1a	1721.3 ± 33.6a	94.15 ± 0.01a	538.7 ± 51.6a	379.0 ± 10.2a
C3	3850	99.67 ± 0.02b	1827.8 ± 18.1a	1768.0 ± 19.72a	89.31 ± 0.04d	543.4 ± 70.4a	364.0 ± 2.7b

^a^Simpson index values had been multiplied by one hundred.

^b^Means followed by different letters within a column indicate significant difference between treatments at *P* < 0.05.

**Fig. 2:**
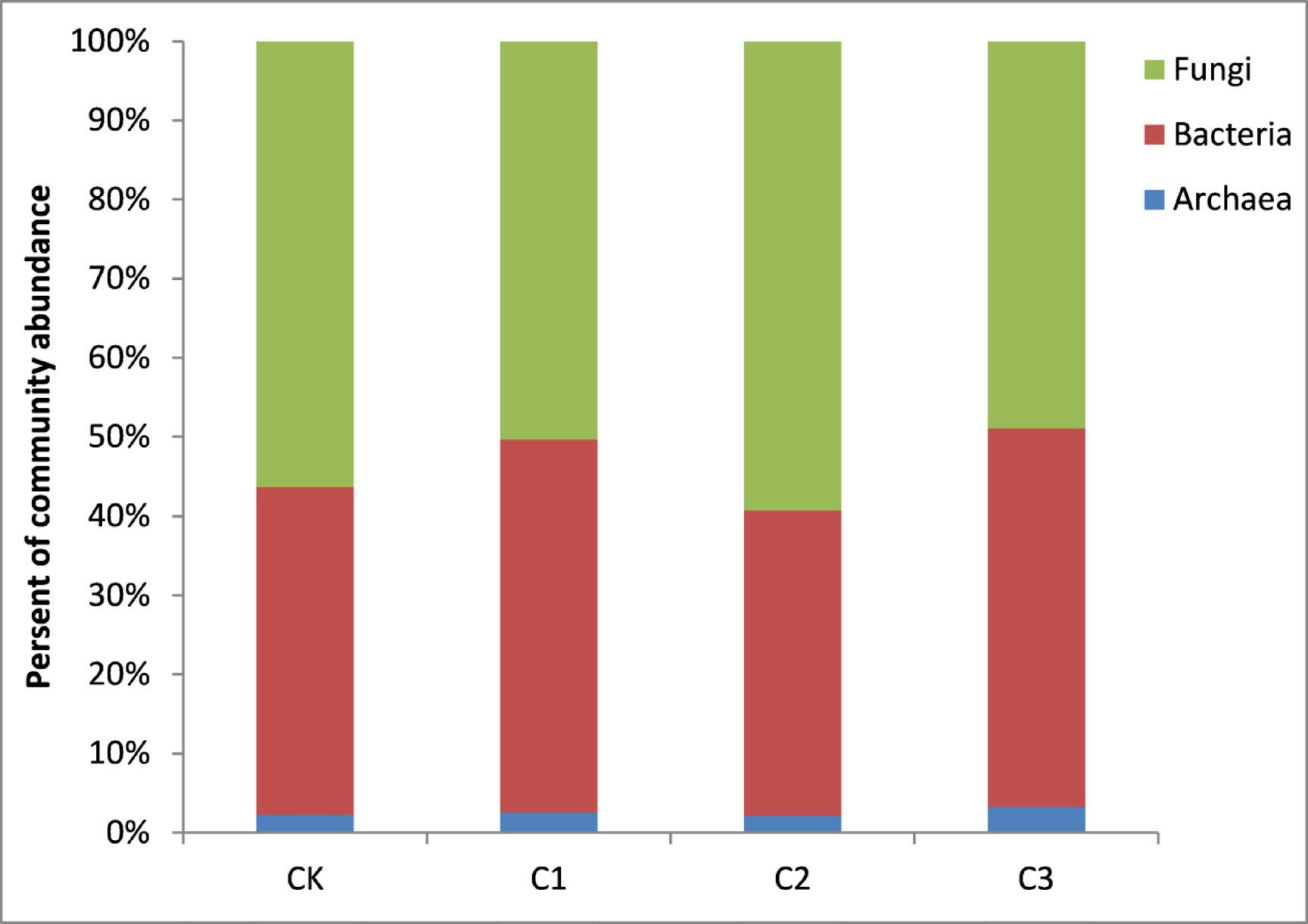
Relative abundances of Archaea, Bacteria and Fungi in different treatments.

**Fig. 3:**
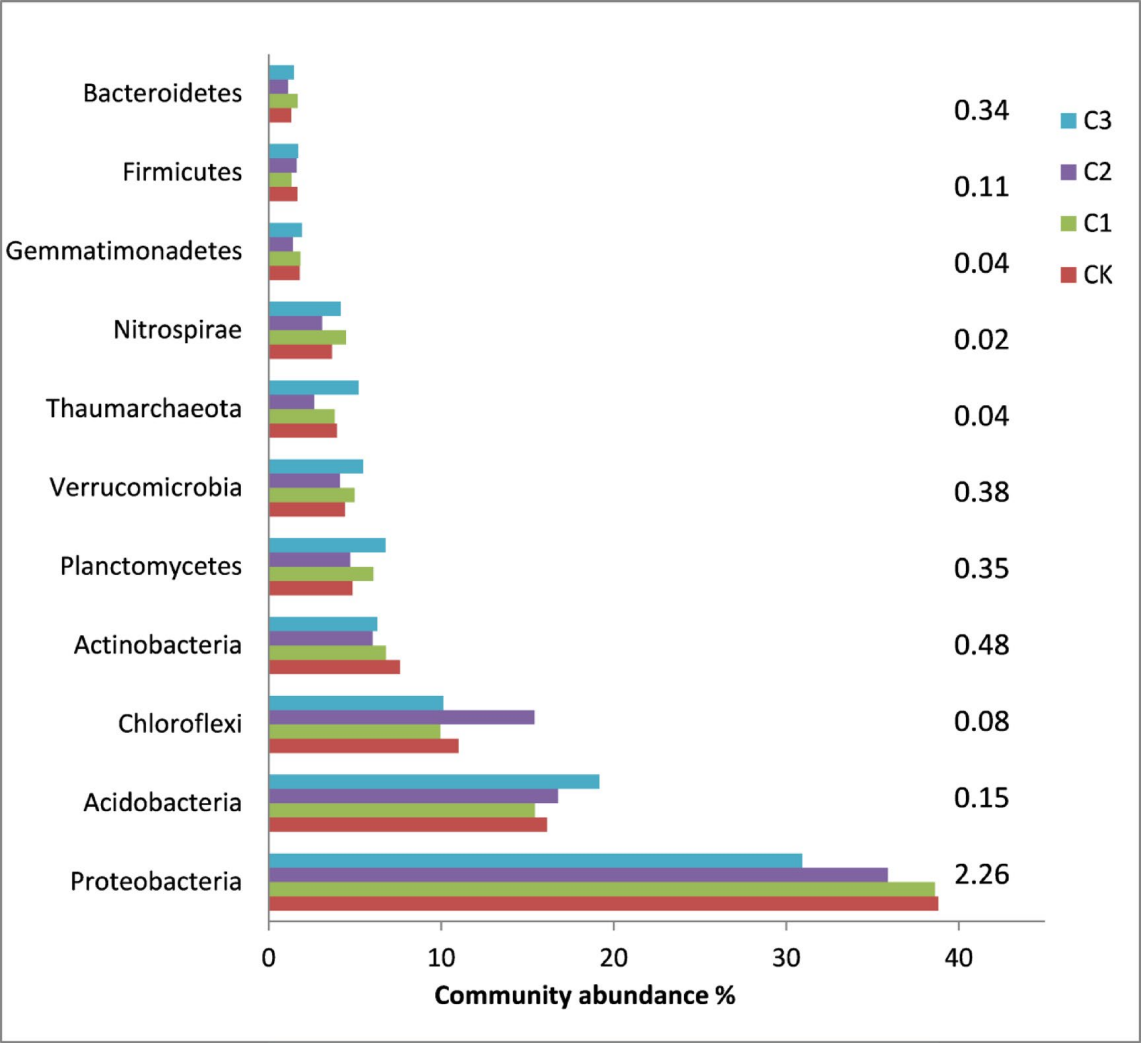
Relative abundances of the dominant soil bacterial phyla in different treatments. *Note*: The *P* value < 0.05(date in the right) indicate significant differences among treatments.

**Fig. 4:**
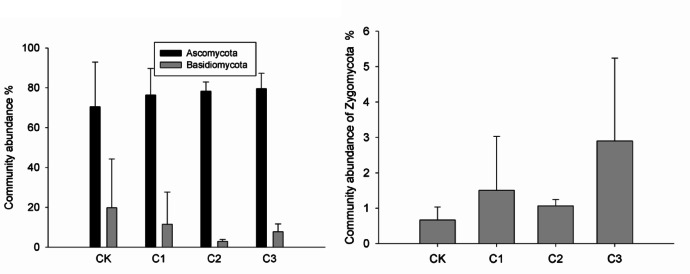
Relative abundances of the dominant soil fungal phyla in different treatments.

**Fig. 5:**
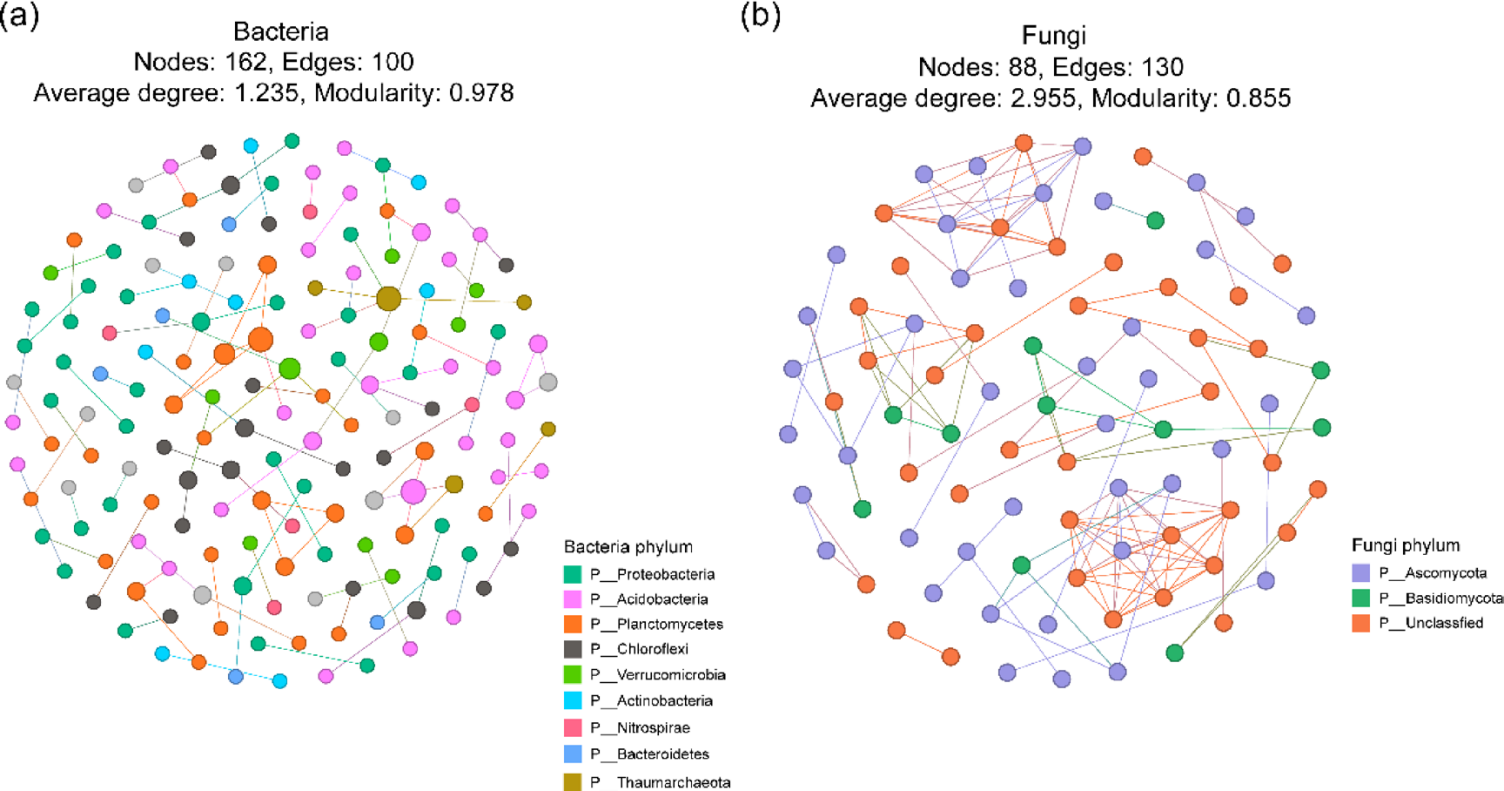
Network of soil bacterial and fungal communities.

The relative abundance of Archaea ranged from 4.48% to 6.11% and increased with increasing biochar dosage (Fig. 2). The dominant bacterial phyla observed in all treatments included Proteobacteria, Acidobacteria, Chloroflexi, Actinobacteria, Planctomycetes, Verrucomicrobia, Thaumarchaeota and Nitrospirae, representing more than 80% of the total bacterial abundance (Fig. 3). Biochar addition tended to increase the abundances of Acidobacteria and Nitrospirae but decreased that of Proteobacteria, especially Deltaproteobacteria. A higher biochar dosage led to a higher relative abundance of Acidobacteria and a lower relative abundance of Proteobacteria in soil. The dominant fungal phyla in all treatments were Ascomycota, Basidiomycota, and Zygomycota (Fig. 4). Biochar application increased the relative abundances of Ascomycota and Zygomycota but decreased that of Basidiomycota. Ascomycota were dominant in CK with a relative abundance of 70.52%, which increased to 79.61% in C3 (Fig. 4 left). Compared to CK, the relative abundance of Basidiomycota was much lower in C2 and C3. The relative abundance of Zygomycota was 0.67% in CK but increased significantly to 2.9% in C3 (Fig. 4 right). The network analysis (Fig. 5) identified the Acidobacteria, Planctomycetes and Thaumarchaeota as keystone OTUs for bacterial communities and Ascomycota as keystone OTUs for fungal communities.

### Relationships between environmental factors and soil bacterial and fungal communities

**Fig. 6:**
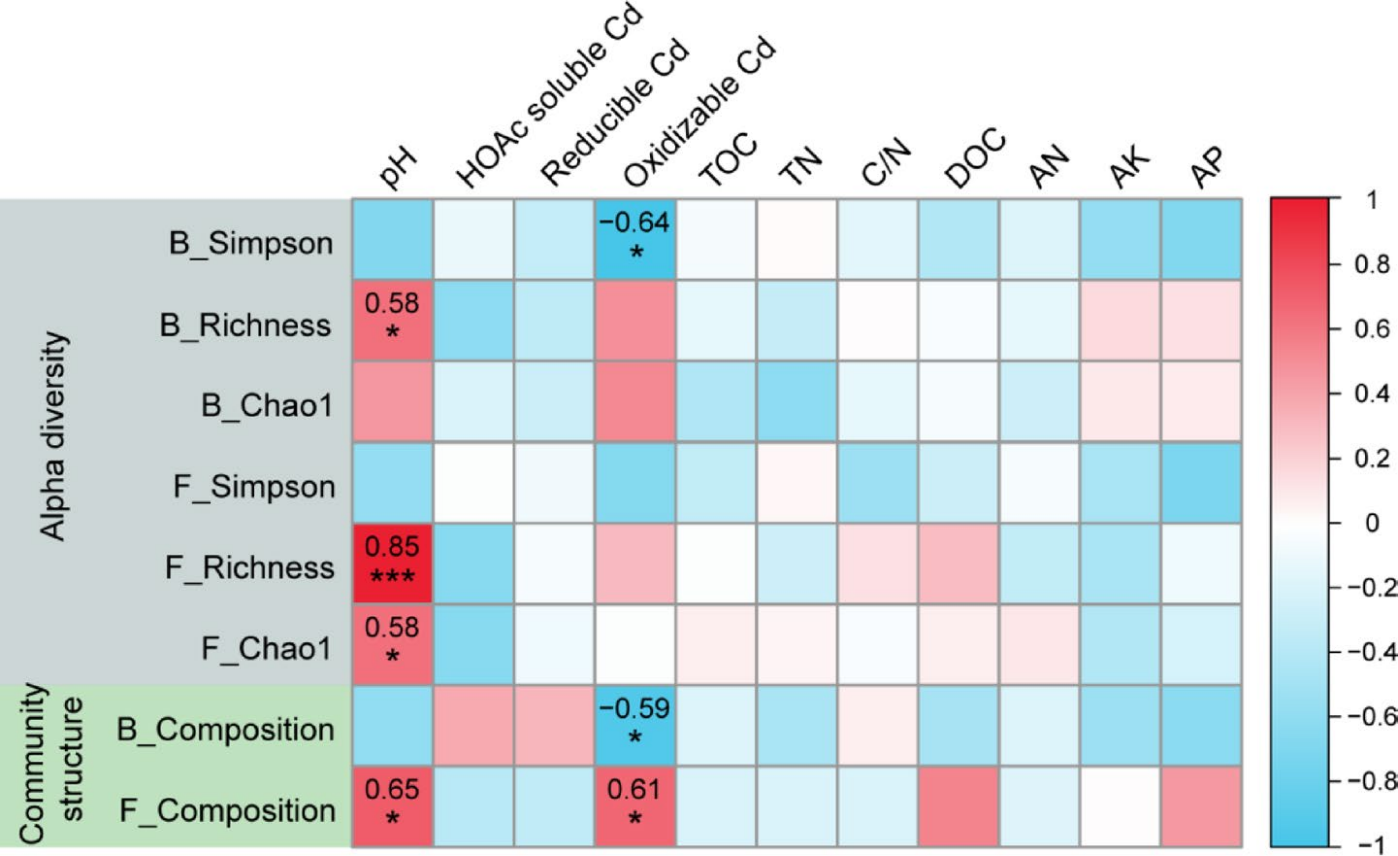
Correlation between microbial diversity and environmental factors. *Note*: Diversity of bacterial and fungal communities (simpson, richness, chao1 index and beta diversity) were used.

**Fig. 7:**
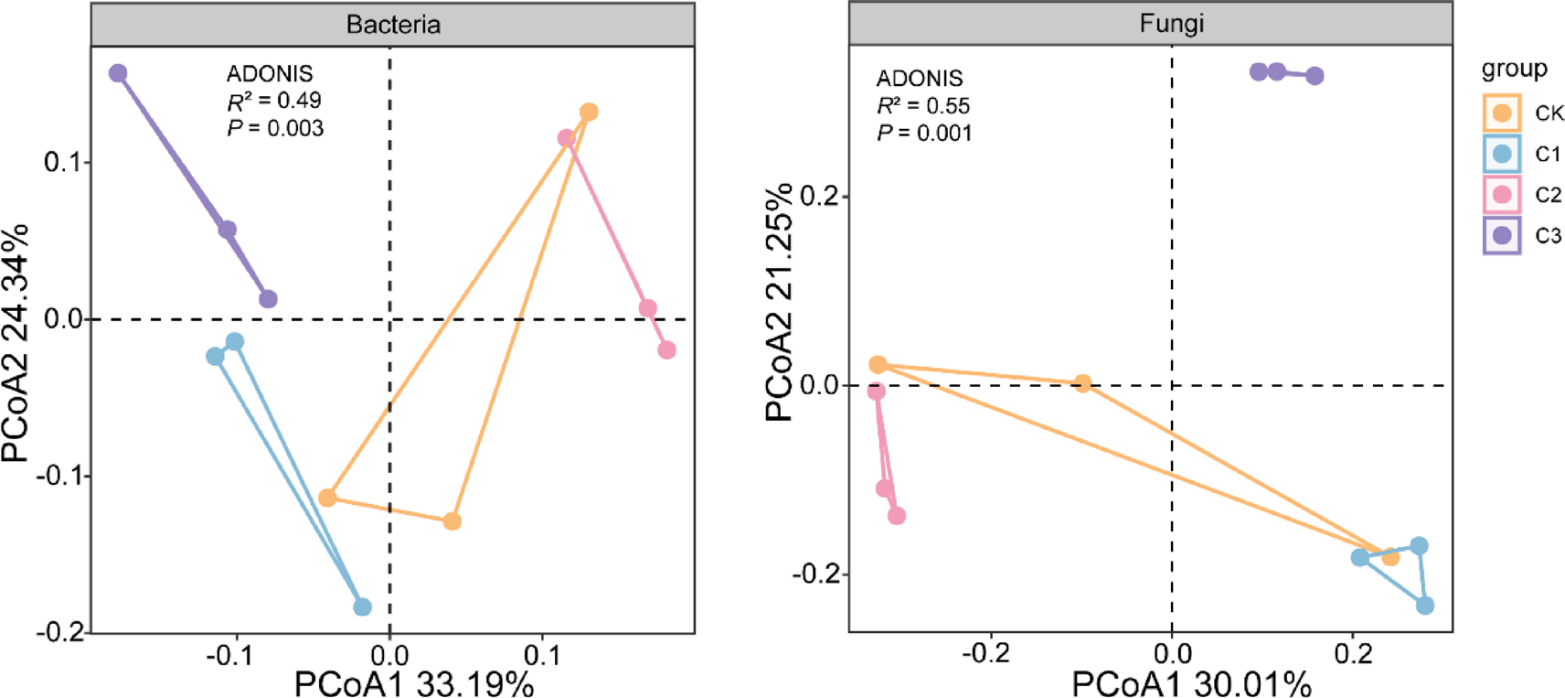
PCoA analysis of bacterial (left) and fungal (right) community composition at the OTU level in the control (CK) and biochar application treatments.

**Fig. 8:**
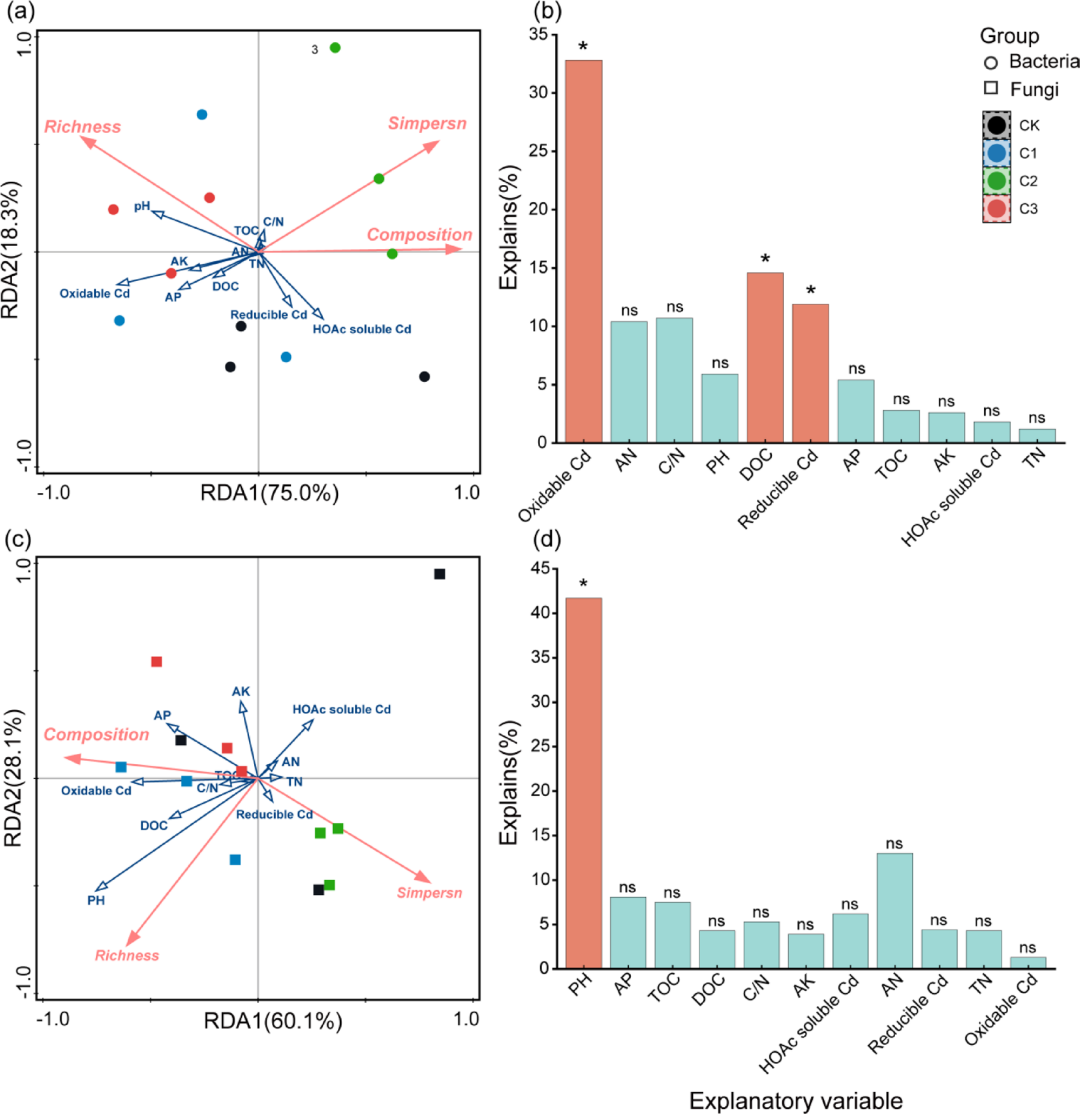
RDA analysis showing the correlations between bacterial (left) and fungal (right) community structures at the OUT level and environmental factors. *Note*: The correlations are indicated by the lengths and angles of the arrows.

Soil bacterial diversity was significantly positively correlated with soil pH and negatively correlated with oxidizable Cd fraction. Soil fungal diversity was positively correlated with soil pH and oxidizable Cd fraction (*P* < 0.05, Fig. 6). Constrained principle coordinate analysis (CPCoA) showed that that microbial communities strongly clustered according to biochar application level, which 57.53% of bacterial abundance variation could be explained by the first two principle components (Fig. 7 left), and 51.26% of fungal abundance variation could be explained by the first two principle components (Fig. 7 right). RDA analysis showed that the environmental factors (soil pH, nutrient contents, and Cd content) had significant influences on soil bacterial and fungal community composition and diversity. Soil oxidizable Cd, DOC, reducible Cd, AN, C/N explained 81% of the total variance in bacterial community composition at OTUs level (Fig. 8). Soil DOC, reducible Cd,and oxidizable Cd contents had significant influences on the soil bacterial community in the biochar application treatments. Soil AN and C/N had relative strong effects on the soil bacterial community in the biochar application treatments as well. In contrast, soil pH had strongest effects on the soil fungal community (Fig. 8), following by AP and TOC.

## Discussion

### Soil microbial community responses to the changes in environmental factors

The results showed that the increase in soil TOC content directly led to the decrease in soluble Cd content. In contrast, the AP change caused by biochar application was the major contributor to the changes in soil reducible and oxidizable Cd fractions. The bacterial community structure was more influenced by AK and oxidizable Cd than by pH and DOC. The fungal community was more sensitive to soil pH and DOC changes than nutrient and Cd content changes. The results suggested that biochar application changed the soil environment and in turn microbial activities, as well as soil soluble Cd content. The changes both in bacterial and fungal community structures were mainly caused by biochar amendment in this study. The different responses of bacteria and fungi to biochar application and the changed environment factors in the study were consistent with other studies. The results implied that biochar indirectly influenced the soil bacteria but directly influenced soil fungi^27–29^. By employing meta-analysis and structural equation modeling analysis, Xu et al.^23^ found that biochar dosage had direct effects on fungal abundance but indirectly influenced bacterial abundance, which was more affected by environmental factors. Furthermore, fungal abundance was the essential factor influencing the response of bacterial abundance to biochar application. Li et al.^2,30^ found available potassium and phosphorus were important environmental factors influencing soil bacterial community. Biochar is generally rich in potassium and phosphorus. Zhang et al.^31^ found that the amount of chemical potassium fertilizer needed was reduced by 40% when biochar was applied at 2%. Biochar accelerates soil potassium availability by changing the clay minerals composition (such as increasing the illite proportion in soil) and promoting the growth of potassium-dissolving bacteria.

### Effects of biochar on soil microbial communities

Our results represented changes of both soil bacterial and fungal community structures within two years since biochar incorporation. Biochar increases microbial metabolic activity in heavy metal-contaminated soils. The biochar-amended soils showed higher Chao diversity than CK treatment. The increased microbial diversity is essential in maintaining the sustainable productivity of rice paddy soils. Yuan et al.^32^ reported that biochar application resulted in higher utilization efficiencies of carbohydrates and carboxylic, α-ketoglutaric, and citric acids by microorganisms, which is of significance for the sustainable productivity of soils as well.

The predominant bacterial phyla in in all treatments were Acidobacteria, Chloroflexi, Actinobacteria, Alphaproteobacteria, and Betaproteobacteria (Fig. 3). Biochar is carbon-rich and assembles organic particles in soils. Two years after biochar application, the relative abundance of Acidobacteria was increased. Generally, Acidobacteria increase and become dominant in biochar-amended soils, which is associated with low SOC mineralization rates^21,33^. They observed that acidobacteria were the predominate rhizobacteria after biochar application, which might because specific microbial communities were assembled by plants to degrade organic matter in biochar. Different changes in the abundance of Deltaproteobacteria after biochar application have been reported in the literature. Significant decrease in Deltaproteobacteria was found in this study, which agreed well with the findings also from rice paddies by other researchers^13,14^. Deltaproteobacteria are iron reducers and exhibit strong remediation abilities for heavy metal and organic pollution. In contaminated soils with biochar, Delta-proteobacteria could constitute a microbial barrier in the rhizosphere, which reduces Cd absorption of rice plants^21^.

In line with the results of previous studies, Ascomycota were the dominant fungal phylum, followed by Basidiomycota and Zygomycota (Fig. 3)^23^. Zheng et al.^13^ reported that biochar application decreased the relative abundance of Ascomycota but greatly increased that of Zygomycota, suggesting fungal community compositions response sensitivity on biochar. Basidiomycota decreased in the biochar application treatments in this study.

### Effect of biochar on soil Cd transformation

Biochar is rich in mineral nutrients, and it has a porous structure. Therefore, when applied to soil, it improves soil nutrient availability and retention, thereby promoting crop growth^34^. Soil available K, available P, and TOC contents were increased in biochar treatments, which was consistent with the results of other studies^34,35^. In contrast, DOC was increased in C1 but decreased in C2 and C3, which may be related to the adsorption and fixation of DOC in soil applied with high dosage of biochar^35,36^.

Two years after biochar application, Cd accumulation in brown rice was significantly decreased (Table 2), and rice yield tent to increase. We found that biochar increased soil pH value and decreased the soluble Cd fraction (Table 3), leading to lower Cd accumulation in rice in this study, which was consistent with the results of previous research. In general, biochar immobilizes soil heavy metals directly through cation exchange processes, adsorption and complexation, as well as indirectly by increased soil pH^4,34,37^. Biochar application increases soil pH due to basic cation releasing, and the soil pH would decrease with biochar ageing^9,38^. Therefore, biochar is more suitable for heavy metal immobilization in acid soils^39–41^. Beside physical and chemical effect of biochar application promote the HOAc soluble Cd fraction transformation into oxidizable and residual Cd fraction, microbial carbonate precipitation also contributed to Cd immobilization^42^, which enhance formations of carbonate bound, Fe–Mn oxides bound, and residual Cd, thereby reducing Cd bioavailability. Wang et al.^21^ demonstrated *Desulfovibrionales* and *Desulfobacterales* preferentially colonized the rice rhizosphere with biochar, which were related to Cd immobilization, and contributed to significantly reducing Cd uptake by rice. Huang et al.^43^ demonstrated that Cd immobilized bacteria, such as *Candidatus_Tenderia* and *Sideroxydans,* were enriched in paddy soils with three-year biochar amendment. This microbial enrichment facilitated the formation of organic matter-bound Cd fractions and effectively reduced the bioavailability of Cd in soil.

## Conclusion

This study demonstrated that biochar amendment could transform soil Cd into oxidizable and residual fractions, and decrease their HOAc soluble fractions. Soil oxidizable Cd, DOC and reducible Cd were important environmental factors causing changes in bacterial communities in the biochar-amended contaminated paddy field. Soil pH was important for fungal communities in the biochar-amended contaminated paddy field. The findings of this study provide insights to different responses between bacteria and fungi on the soil environment changed by biochar amended in long-term. Bacterial community was sensitive to Cd immobilization and DOC fluctuation. Fungal community was mainly influenced by changed soil pH. Biochar application successful stabilization of Cd in contaminated soils and changed microbial compositions.

## Electronic supplementary material

Below is the link to the electronic supplementary material.

Supplementary Material 1

## Data Availability

Sequence data of this study were deposited into the NCBI Sequence Read Archive (SRA) database (Accession numbers: SRR2146924 and SRR2146952).
