# Supplementary material for: Soil bacterial and fungal communities respond differently to the biochar amendment in a cadmium-contaminated paddy field
Source: Sci Rep. 2025 Oct 14;15:35764. doi: 10.1038/s41598-025-02182-w (PMC12521584; doi:10.1038/s41598-025-02182-w)
Supplement: Supplementary file 1 — Supplementary Material 1 [file 41598_2025_2182_MOESM1_ESM.docx]

***DNA extraction and Illumina MiSeq sequencing***

Microbial community genomic DNA was extracted from the soil samples using the EZNA^®^ soil DNA kit (Omega Bio-tek, Norcross, GA, USA) according to the manufacturer’s instructions. The quality and concentration of DNA were determined using 1.0% agarose gel electrophoresis and a NanoDrop® ND-2000 spectrophotometer (Thermo Scientific Inc., USA), respectively. The V4 region of the bacterial 16S rRNA gene was amplified using the primer pair 515F (5'-GTGYCAGCMGCCGCGGTAA-3') and 806R (5'-GGACTACNVGGGTWTCTAAT-3'), and the fungal ITS gene was amplified using the primer pair 1737F (5'-GGAAGTAAAAGTCGTAACAAGG-3') and 2043R (5'-GCTGCGTTCTTCATCGATGC-3') (Zhang et al. 2016). Purified amplicons were pooled in equimolar amounts and paired-end sequenced on an Illumina MiSeq platform (Illumina, San Diego, USA) according to the standard protocols provided by Majorbio Bio-Pharm Technology Co., Ltd. (Shanghai, China). The bacterial and fungal community compositions in soil were determined by Illumina Miseq sequencing. The raw reads were deposited into the NCBI Sequence Read Archive (SRA) database (Accession numbers: SRR2146924 and SRR2146952).

The raw FASTQ files were demultiplexed and subsequently quality-filtered using fastp version 0.19.6 (Chen et al. 2018). The merging process was performed using FLASH version 1.2.7. Reads were trimmed at positions where the average quality score was below 20 within a moving 50-bp window. Additionally, any reads that became shorter than 50 bp after trimming were removed. Reads containing ambiguous characters were also discarded. Only overlapping sequences that were longer than 10 bp were assembled based on their overlapping segments. The maximum allowable mismatch ratio in the overlap region was set at 0.2. Samples were differentiated based on the barcodes and primers used, and sequence orientation was adjusted accordingly. Adjustments involved exact barcode matching and allowed for a two-nucleotide mismatch in primer matching. Subsequently, the optimized sequences were grouped into operational taxonomic units (OTUs) using UPARSE 7.1, with the sequence similarity threshold set at 97% (Edgar 2013). The most abundant sequence within each OTU was chosen as the representative sequence. Taxonomic classification of these representative sequences from each OTU was performed using RDP Classifier version 2.2 against the 16S rRNA gene database, with a confidence threshold of 0.7.

**References**

Zhang, L., Zhang, H., Wang, Z., Chen, G., & Wang, L. (2016). Dynamic changes of the dominant functioning microbial community in the compost of a 90-m.(3). aerobic solid state fermentor revealed by integrated meta-omics. *Bioresource Technology, 203*, 1-10. https://doi.org/10.1016/j.biortech.2015.12.040

Edgar, R. C. (2013). UPARSE: highly accurate OTU sequences from microbial amplicon reads. *Nature Methods, 10,* 996-998. https://doi.org/ 10.1038/nmeth.2604

Chen, S., Zhou, Y., Chen, Y., & Gu, J. (2018). Fastp: an ultra-fast all-in-one FASTQ preprocessor. *Bioinformatics, 34*, 884-890. https://doi.org/10.1093/bioinformatics/bty560
